# Synthetic Physical Interactions with the Yeast Centrosome

**DOI:** 10.1534/g3.119.400117

**Published:** 2019-05-10

**Authors:** Rowan S. M. Howell, Attila Csikász-Nagy, Peter H. Thorpe

**Affiliations:** * The Francis Crick Institute, London, NW1 1AT UK; † Randall Division of Cell and Molecular Biophysics, King’s College, London, SE1 1UL UK; ‡ Faculty of Information Technology and Bionics, Pázmány Péter Catholic University, Budapest, 1083 Hungary; § School of Biological and Chemical Sciences, Queen Mary University, London, E1 4NS UK

**Keywords:** centrosome, localization, Spindle Pole Body, empirical Bayes

## Abstract

The yeast centrosome or Spindle Pole Body (SPB) is an organelle situated in the nuclear membrane, where it nucleates spindle microtubules and acts as a signaling hub. Various studies have explored the effects of forcing individual proteins to interact with the yeast SPB, however no systematic study has been performed. We used synthetic physical interactions to detect proteins that inhibit growth when forced to associate with the SPB. We found the SPB to be especially sensitive to relocalization, necessitating a novel data analysis approach. This novel analysis of SPI screening data shows that regions of the cell are locally more sensitive to forced relocalization than previously thought. Furthermore, we found a set of associations that result in elevated SPB number and, in some cases, multi-polar spindles. Since hyper-proliferation of centrosomes is a hallmark of cancer cells, these associations point the way for the use of yeast models in the study of spindle formation and chromosome segregation in cancer.

Microtubule Organizing Centres (MTOCs) are critical to the process of chromosome segregation in eukaryotes. Abnormalities in the structure or number of centrosomes, the metazoan MTOC, are strongly associated with human cancer (Nigg 2006). In *S. cerevisiae*, the MTOC is the Spindle Pole Body (SPB). The SPB differs from metazoan centrosomes in its structure and in that it remains embedded in the nuclear membrane throughout the closed mitosis of yeast (Fu *et al.* 2015). However, despite these differences, there is significant conservation between yeast SPB proteins and human centrosomal proteins (Jaspersen and Winey 2004), making the yeast SPB a relevant model of MTOCs.

Beyond their roles in microtubule nucleation, SPBs are thought to act as signaling hubs, with recruitment to the SPB a key step in regulation of certain signaling pathways (Fu *et al.* 2015; Arquint *et al.* 2014). Various studies have used the strong interaction between GFP and GFP-Binding Protein (GBP) (Rothbauer *et al.* 2006), to test the effect of forced localization to the SPB, for example (Gryaznova *et al.* 2016; Caydasi *et al.* 2017). However, no systematic study of forced relocalization to the SPB has been performed. We used the Synthetic Physical Interaction (SPI) methodology (Ólafsson and Thorpe 2015) to test recruitment of more than 4,000 proteins to five locations around the SPB.

Proteome-wide SPI screens have been used in the past to probe the regulation of the kinetochore (Ólafsson and Thorpe 2015, 2016) and a set of 23 SPI screens was used to generate a cell-wide map of proteins sensitive to relocalization (Berry *et al.* 2016). Relative to the screens of Berry *et al.* our analysis shows that the SPB is particularly sensitive to forcible relocalization. As a result, we found that standard methods for analysis of genome-wide screens based on Z-transformations were unsuitable to analyze these screens. Efron (2004) suggested an approach to multiple hypothesis testing, such as genome-wide screens, based around an empirically derived null distribution which he treated as a component of a finite mixture model to calculate significance of measured results. This empirical Bayes approach is widely used to analyze gene expression data, where it is used to classify the significance of correlations between genes, see for example (Schafer and Strimmer 2005). We adapted this approach to the analysis of the 23 SPI screens conducted by Berry *et al.* (2016) as well as the five SPB SPI screens. We fit bimodal normal mixture models to our data, according to an approach outlined in (Fraley and Raftery 2002). This approach overcomes the limitations of Z-transformations as well as providing a parameterisation to compare screens and tools to predict the rate of validation. Berry *et al.* (2016) concluded that only ∼2% of proteins were sensitive to forcible localization, our analysis suggests that locally this may vary, with some regions, such as the SPB, far more sensitive than other parts.

Global analysis of the SPI data shows that the SPB is sensitive to forced interactions with a variety of proteins, including proteins involved in microtubule nucleation, protein transport, lipid biosynthesis and the cell cycle. Proteins that caused growth defects when recruited to the SPB originated from the nucleus and chromosomes as well as membranes, especially the endoplasmic reticulum. Although we found significant variation in individual results between regions of the SPB, the data from the SPB SPI screens was found to be more similar to each other than to the screens with other parts of the cell. A particularly interesting finding is that tethering nuclear pore proteins to the SPB causes growth defects. A growing body of work (reviewed in Jaspersen and Ghosh (2012); Rüthnick and Schiebel (2018)), argues that the process of SPB duplication and insertion into the nuclear membrane relies on machinery usually associated with the nuclear pore. We investigated whether these forced interactions between Spc42 and nuclear pore proteins resulted in abnormal SPB number. We found that forced recruitment of several nuclear pore proteins, as well as the SPIN (SPB Insertion Network) and some currently-unclassified membrane proteins showed evidence of SPB overduplication. The current model for SPB duplication is that it is tied to the cell division cycle through sequential activation by Cdc14 and CDK (Rüthnick and Schiebel 2018). Our work suggests that forced localization of proteins to the SPB can decouple the process of SPB duplication from the cell cycle, a finding that may suggest refinement of the current model or that SPB duplication can occur via alternative pathways. The development of yeast strains that reliably produce multi-polar spindles may facilitate research into these structures which are known to occur in cancer cells which exhibit high variability in centrosome number. (Nigg 2006).

## Materials and Methods

### Yeast strains and methods

Yeast was cultured in standard growth media with 2% (w/v) glucose unless otherwise stated. GFP strains in this study are from a library derived from BY4741 (his3Δ1 leu2Δ0 met15Δ0 ura3Δ0) (Huh *et al.* 2003; Tkach *et al.* 2012). For each screen we constructed plasmids expressing an SPB-GBP-RFP construct and the SPB protein alone from the *CUP1* promoter; all plasmids are derived from pWJ1512 (Reid *et al.* 2011) and are listed in Table 1. Plasmids were constructed by gap repair either through *in vivo* recombination or the NEBuilder plasmid assembly tool (New England Biolabs, USA). Linear products were created by PCR with primers from Sigma Life Science and Q5 Polymerase (New England Biolabs, USA). The sequence of azurite fluorescent protein (Mena *et al.* 2006) was synthesized by GeneArt (ThermoFisher Scientific, UK). To visualize spindle morphology, Bbp1-, Nup170-, Nup133- and YJL021C-YFP mTurq-TUB1 strain were constructed using a linear construct to convert GFP to YFP and a linearized plasmid, “pHIS3p:mTurquoise2-Tub1+3’UTR::URA3”, which was a gift from Wei-Lih Lee (Addgene plasmid # 50635; http://n2t.net/addgene:50635; RRID:Addgene_50635) (Markus *et al.* 2015). The sequence of all plasmids was verified by Sanger sequencing (Genomics Equipment Park STP, Francis Crick Institute and Genewiz, UK).

**Table 1:** Table of plasmids

Plasmid Name	Genotype	Selection
pHT4	*GBP-RFP*	Leu\Amp
pHT11	*SPC42-GBP-RFP*	Leu\Amp
pHT222	*SPC42-RFP*	Leu\Amp
pHT297	*SPC42*	Leu\Amp
pHT575	*SPC110-GBP-RFP*	Leu\Amp
pHT576	*GBP-RFP-SPC110*	Leu\Amp
pHT577	*SPC110*	Leu\Amp
pHT584	*NUD1-GBP-RFP*	Leu\Amp
pHT585	*NUD1*	Leu\Amp
pHT615	*SPC72*	Leu\Amp
pHT616	*SPC72-GBP-RFP*	Leu\Amp
pHT706	*HTB2-AZURITE*	Nat\Amp

### SPI screening

The SPI screening process is described in detail in Berry *et al.* (2016) and in Ólafsson and Thorpe (2018). A library of GFP strains is transformed with a plasmid expressing either a fusion of a protein of interest with GBP or a control, through a mating-based method known as Selective Ploidy Ablation (SPA) (Reid *et al.* 2011). The plates are repeatedly copied and grown on successive rounds of selection media until a library of haploid GFP strains with the plasmid is produced. This library is assayed for colony size, giving a readout for the fitness of a given binary fusion between the GFP strain and protein of interest. Plates were scanned on a desktop flatbed scanner (Epson V750 Pro, Seiko Epson Corporation, Japan) at a resolution of 300 dpi. All plates were grown at $30^{\circ }$C. All copying of yeast colonies was performed on a Rotor robot (Singer Instruments, UK).

### Quantitative analysis of high-throughput yeast growth

Scanned images were analyzed computationally to extract measurements of the colony sizes. The online tool ScreenMill (Dittmar *et al.* 2010) was used to perform normalization and calculate Log Growth Ratios (LGRs) and Z-scores by comparison of experimental and control colony sizes. Two controls were used (plasmids expressing GBP or the SPB protein alone) but, as in previous studies, we found strong agreement between the two and we used an average of the two values. In some cases, the library contained multiple copies of the same GFP strain, in these cases data were aggregated by averaging. In the proteome-wide screens plates were normalized to the plate median while in the validation screens GFP-free controls were used for normalization. LGRs were further normalized using a spatial smoothing algorithm as described in Berry *et al.* (2016). Bimodal normal mixture models were fitted to the smoothed LGR data using the “Mclust” package (Scrucca *et al.* 2016). Further details can be found in the supplementary materials. R scripts for data formatting and analysis are freely available at https://github.com/RowanHowell/data-analysis.

### Bioinformatics

The GOrilla website (cbl-gorilla.cs.technion.ac.il (Eden *et al.* 2009)) was used to perform all gene ontology enrichment analysis. The “cluster” program (version 3.0) (Eisen *et al.* 1998) was used to perform hierarchical clustering of the SPI data; Java Treeview (Saldanha 2004) was used to visualize the results. Clustering was performed using the correlation of the LGRs, minimizing the average linkage of the clusters. Spatial Analysis of Functional Enrichment (SAFE) analysis of SPB SPI hits was performed using the Cell Map website (thecellmap.org (Usaj *et al.* 2017)). The Venn diagram in Supplementary Figure S1 was created at bioinformatics.psb.ugent.be/webtools/Venn/.

### Fluorescence microscopy

To examine localization of the SPB-GBP-RFP construct, and GFP-tagged proteins, cells were grown shaking overnight at $23^{\circ }$C in -leucine media, supplemented with additional adenine. They were then imaged with a Zeiss Axioimager Z2 microscope (Carl Zeiss AG, Germany), with a 63x 1.4NA oil immersion lens and using a Zeiss Colibri LED illumination system (RFP = 590 nm, YFP = 505 nm, GFP = 470 nm, mTurq = 445 nm, azurite = 385nm). Bright field images were obtained and visualized using differential interference contrast (DIC) prisms. Images were captured using a Hamamatsu Flash 4 Lte. CMOS camera containing a FL-400 sensor with 6.5 mm pixels, binned 2x2. Images were prepared with Volocity software (Perkin Elmer Inc., USA). To screen for abnormal numbers of foci in strains containing Spc42-GBP-RFP and GFP-tagged proteins, a plate of strains was prepared using the SPA methodology described above. In this assay, the Spc42-GBP-RFP plasmid (pHT11) was accompanied by a plasmid expressing Htb2-Azurite (pHT 706) with nourseothricin (*NAT*) selection. The Htb2-Azurite construct allowed for identification of the nucleus. On the same day, cells were picked from the plate and suspended in water and them imaged as described above. Dead cells were identified by a high level of dispersed fluorescence, and were excluded, as were cells with no visible fluorescence in the RFP channel.

### Data availability

Plasmid details are shown in Table 1, all strains are available on request. File S1 contains data from the colocalization assay. File S2 contains all LGRs from SPI screens used in this study. File S3 contains all LGRs from validation of SPB SPI screens. File S4 contains a list of hits from all screens used in this study, based on either the empirical Bayes method or Z-score where appropriate. File S5 contains full results of GO enrichment analysis. Table S1 contains a list of all empirical Bayes parameters and cutoffs used for screens in this study. Table S2 contains a comparison of empirical Bayes parameters fitted over entire GFP library and restricted to GB library for relevant screens used in this study. Table S3 contains the data from the supernumerary RFP foci screen. Table S4 contains the data for quantification of the supernumerary RFP foci phenotype. Supplemental material available at FigShare: https://doi.org/10.25387/g3.8100749.

## Results

### Synthetic Physical Interaction screens with the SPB

**Figure 1:**
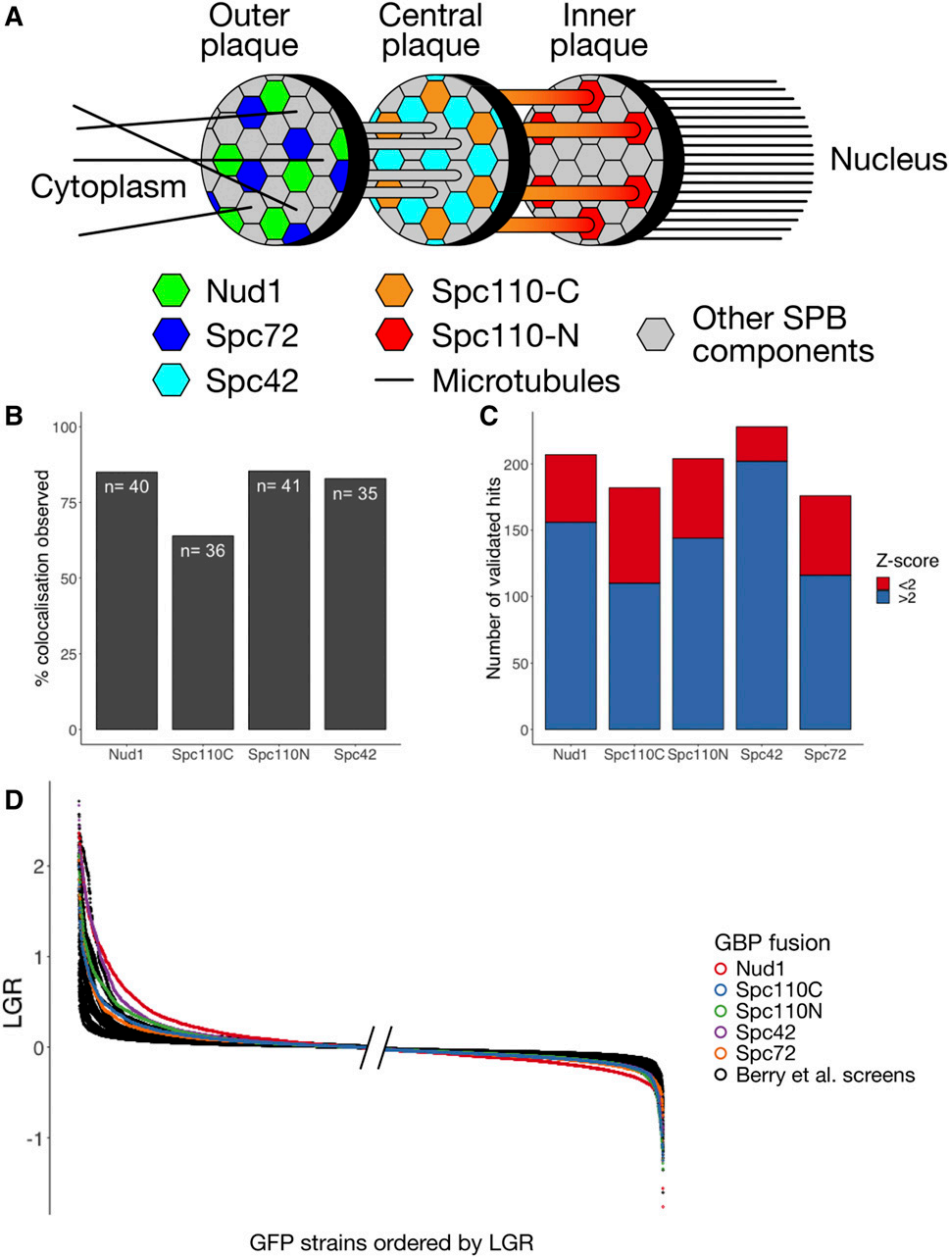
A: Structure of the SPB showing location of GBP tags used. B: Colocalization of query and target proteins in the Nud1, Spc42, Spc110C and Spc110N screens. A selection of 48 GFP strains was chosen to represent different regions of the cell and a mixture of strong and weak growth phenotypes. Each strain was judged to have either colocalization of GFP and RFP at SPB foci or not. In some cases no live cells were imaged due to slow growth, these strains were removed from analysis. The 60%−80% colocalization observed in each screen is consistent with previous studies (Berry *et al.* 2016). C: Validation of SPB SPI screens. For each GBP construct, 240 GFP strains were chosen and rescreened at higher density. These strains were considered to be validated hits if the growth defect measured was greater than a cutoff determined by GFP-free controls. In each screen, we found that strains with Z-scores less than 2 met the criteria for validation, suggesting the cutoff at a Z-score of 2 was overly restrictive. D: Ordered LGRs for each of the 5 SPB screens and 23 screens from Berry *et al.* (2016), this graph shows only strains present in the subset of the GFP library used in the SPB screens. The left hand side of the graph has left-justified values while the right-hand side shows the right-justified values, this is because the region closest to the edges is the most informative. The SPB screens, shown in color, are considerably separated from the screens performed with other regions of the cell.

The budding yeast SPB is embedded in the nuclear membrane with one face, known as the inner plaque, directed into the nucleus and the other, known as the outer plaque, facing into the cytoplasm (Jaspersen and Winey 2004) (Figure 1A). A central plaque links the inner and outer faces of the SPB and connects to a structure known as the half-bridge, which is involved in SPB duplication. In order to understand the effect of localizing proteins to different parts of the SPB, we performed genome-wide Synthetic Physical Interaction screens with multiple target proteins: Nud1, Spc42, Spc72 and Spc110 N-termini and Spc110 C-terminus GBP fusions. Nud1 and Spc72 are situated in the outer plaque of the SPB; the N-terminus of Spc110 lies on the inner plaque while its C-terminus is located, with Spc42, in the central plaque (Jaspersen and Winey 2004).

SPI screens aim to test the effects of forced relocalization of gene products across the genome (Ólafsson and Thorpe 2015). In each screen, a target gene tagged with GBP (GFP-Binding Protein) is introduced into a library of GFP strains (Tkach *et al.* 2012) to induce binary fusions between the target protein and the GFP-tagged query protein. Growth of colonies under these conditions is measured and an average LGR (Log Growth Ratio) between the experimental strain and two control strains is calculated, providing a measure of any growth defect caused by the artificial protein-protein interaction. Additionally, a Z-transformation is applied to assess the significance of the results. A Z-transformation assumes the data are normally distributed and uses the mean and variance of the data to transform each data point to a Z-score, which are distributed according to a normal distribution with a mean of 0 and a standard deviation of 1. This simplifies analysis, in Z-space the region (−2,2) represents the 95% confidence interval, so a Z-score greater than 2 is indicates a significant deviation from the mean. Similar to genetic interactions, we say a forced association between proteins is a SPI only if this combination causes a significant growth defect.

**Figure 2:**
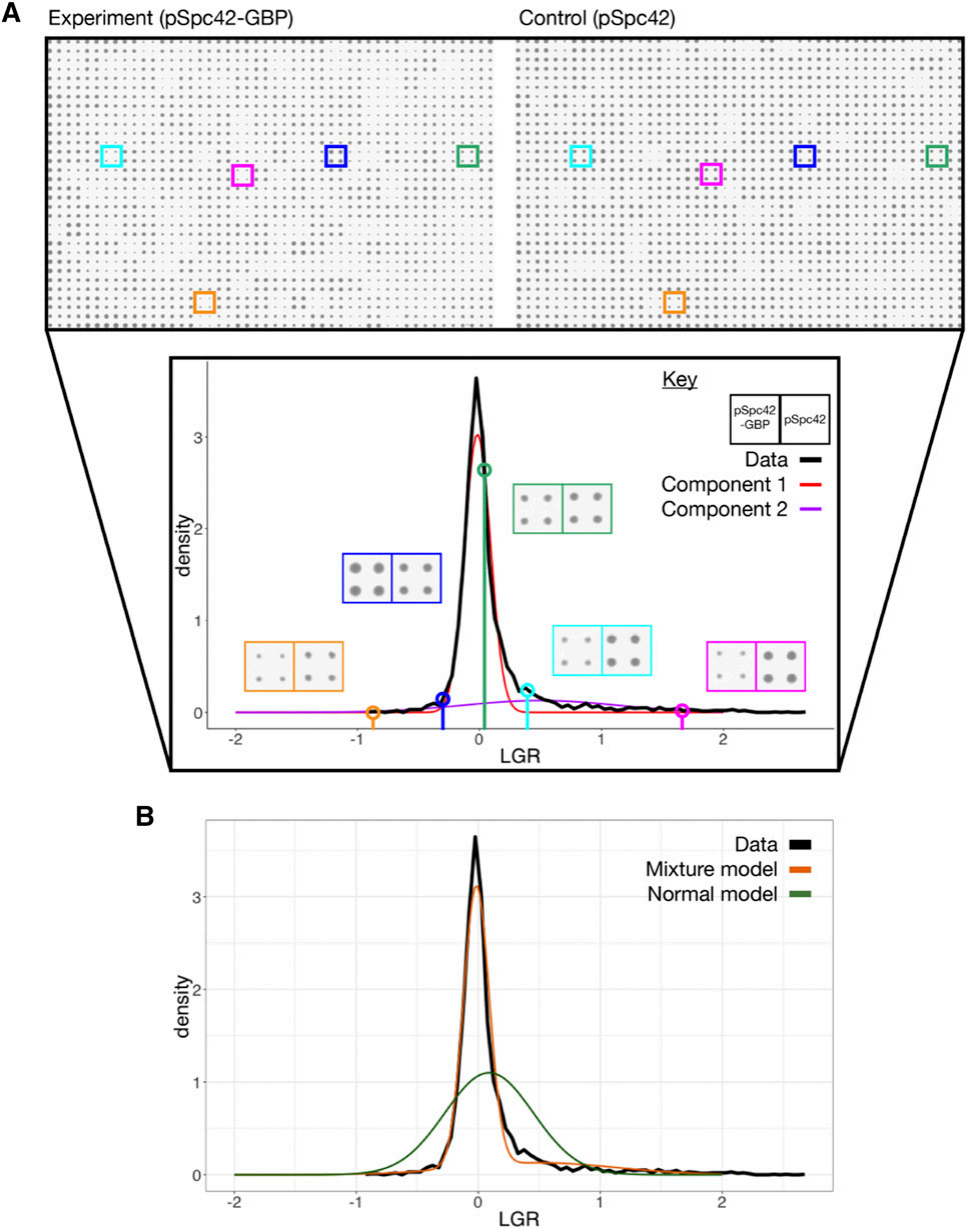
A: Schematic of the mixture model analysis of SPI screen data for the Spc42 screen. The top panel shows scans of a single library plate with the plasmid expressing either Spc42-GBP-RFP (denoted Spc42-GBP) or Spc42 (the plate with the plasmid expressing GBP alone is not shown). The lower panel shows a histogram of LGRs in the screen, with two normal components of the mixture model shown in color. Five strains are highlighted to show the difference in colony size associated with different LGRs. Note that strains with low negative LGRs, such as that shown in orange are often the results of slow-growing GFP strains, which can register as having enhanced growth due to plate normalization and proportionally high levels of measurement error. B: Comparison of the bimodal normal mixture model and normal model of the Spc42 screen data, with the histogram of measured LGRs.

We predicted that, due to the structural integrity of the SPB, the GFP-tagged query proteins were more likely to be recruited to the GBP-tagged target protein than vice versa. We used fluorescence microscopy to analyze colocalization of GFP- and GBP-tagged proteins in 48 GFP strains chosen to represent a mixture of hits and non-hits as well as clearly-defined and distinct regions of the cell as well as regulators of mitosis. We found 60%−80% of strains viewed showed localization patterns consistent with recruitment of the query protein to the SPB (Figure 1B, Supplementary File S1) in the Nud1, Spc42, Spc110C and Spc110N screens; a finding in keeping with the results of Berry *et al.* (2016). Genome-wide screens often have high rates of type I errors (false positives) so we validated a selection of strains with high or, in some cases, low, negative LGRs. Validation screens were performed with 16, rather than 4, replicates of each strain and “validation” of a result was defined by a LGR exceeding a threshold set by GFP-free controls. Each of the screens identified ∼150 strains with Z-scores greater than 2 and we validated 240 strains for each screen. The remaining strains were chosen as those just below the Z-score of 2 cutoff and “growth enhancers” - strains with Z-score less than −2, these were found mainly in the Spc110C and Spc110N screens. The growth enhancers were found not to validate frequently in either screen, these strains are likely slow growing generally, which can lead to inaccurate LGRs (see Figure 2A). We were surprised to discover that almost all of the strains with Z-scores above 2 validated and many that lay below this cutoff validated as well (Figure 1C, Supplementary File S3). Furthermore, when we plotted the distribution of LGRs against LGRs from a dataset of 23 SPI screens (Berry *et al.* 2016), we noticed that the SPB screens generally had more high-LGR strains than other screens (Figure 1D). We hypothesized that the SPB was particularly sensitive to forced localization and that these screens identified many true hits. However this was not reflected in the number of hits according to the Z-score. As the Z-transformation is based on the assumption that data are normally distributed, it will become inappropriate when the data deviates significantly from this distribution, as we would expect in the case of a screen with many hits. Therefore, we developed a novel statistical methodology to analyze significance in SPI screens.

### Mixture models are an effective model for SPI screen data

Genome-wide screens, such as SPI screens, typically apply an experimental procedure to assign every gene in the genome a value. In yeast screens, such as SPI or yeast-two-hybrid screens, this measure often characterizes the growth of a colony. Analysis of these screens generally assumes that the distribution of colony sizes under equal conditions will follow a lognormal distribution, so that the logarithm of colony sizes is normally distributed. However, when performing a genome-wide screen, we expect some small but non-zero proportion of strains to have reduced fitness and grow more slowly. We hypothesized that in certain cases, where a significant number of genes are affected, screening data will not fit a normal distribution. In a previous study, Berry *et al.* (2016) performed 23 SPI screens using GBP fusions in different compartments of the cell, in order to build up a map of protein localization sensitivity. We combined this dataset of screens with the SPB SPI data to assess the performance of Z-transformations in different proteome-wide screens.

We found that the LGRs are not distributed according to a normal distribution (Figure 2B). We reasoned that, similarly to Efron (2004), we could take advantage of the assumption that the data contained two distinct categories, unaffected and affected by forced localization, to develop an improved statistical model of the data (Supplementary Methods). We used the “Mclust” package (Scrucca *et al.* 2016) to fit bimodal normal mixture models (Fraley and Raftery 2002) to the SPI data (Figure 2). These mixture models matched the distribution of SPI data more successfully than unimodal normal distributions (Figure 2B). We found that for 20 of the 28 screens the fitted mixture model matched our intuition of a “central” peak representing unaffected genes and a “hit” peak, shifted to the right representing genes affected by the forced interaction (Supplementary Table S1). The data for the remaining eight screens did not show well-defined hit peaks. An underlying assumption of our analysis is that the non-hits will be distributed according to a normal distribution, so in screens with few hits, we would expect a normal model to fit the data effectively. We interpret the failure of the mixture model to identify a well-defined hit peak in these eight screens as indicating that the screens have few hits and that therefore, in these cases, a Z-transformation would be appropriate. When present, the two overlapping, peaks in the data allows for the identification of two defined categories in the data. Component 1, or the central peak, contains genes unaffected by the interaction and is distributed normally due to noise in measurement. Component 2, or the hit peak, contains genes affected by the interaction, the shape of this distribution represents both effects of noise and the distribution of strength of real growth defects. We do not know *a priori* the shape of the distribution of interactions effects, but here we make the assumption it is Gaussian.

### Tools based on mixture models

**Figure 3:**
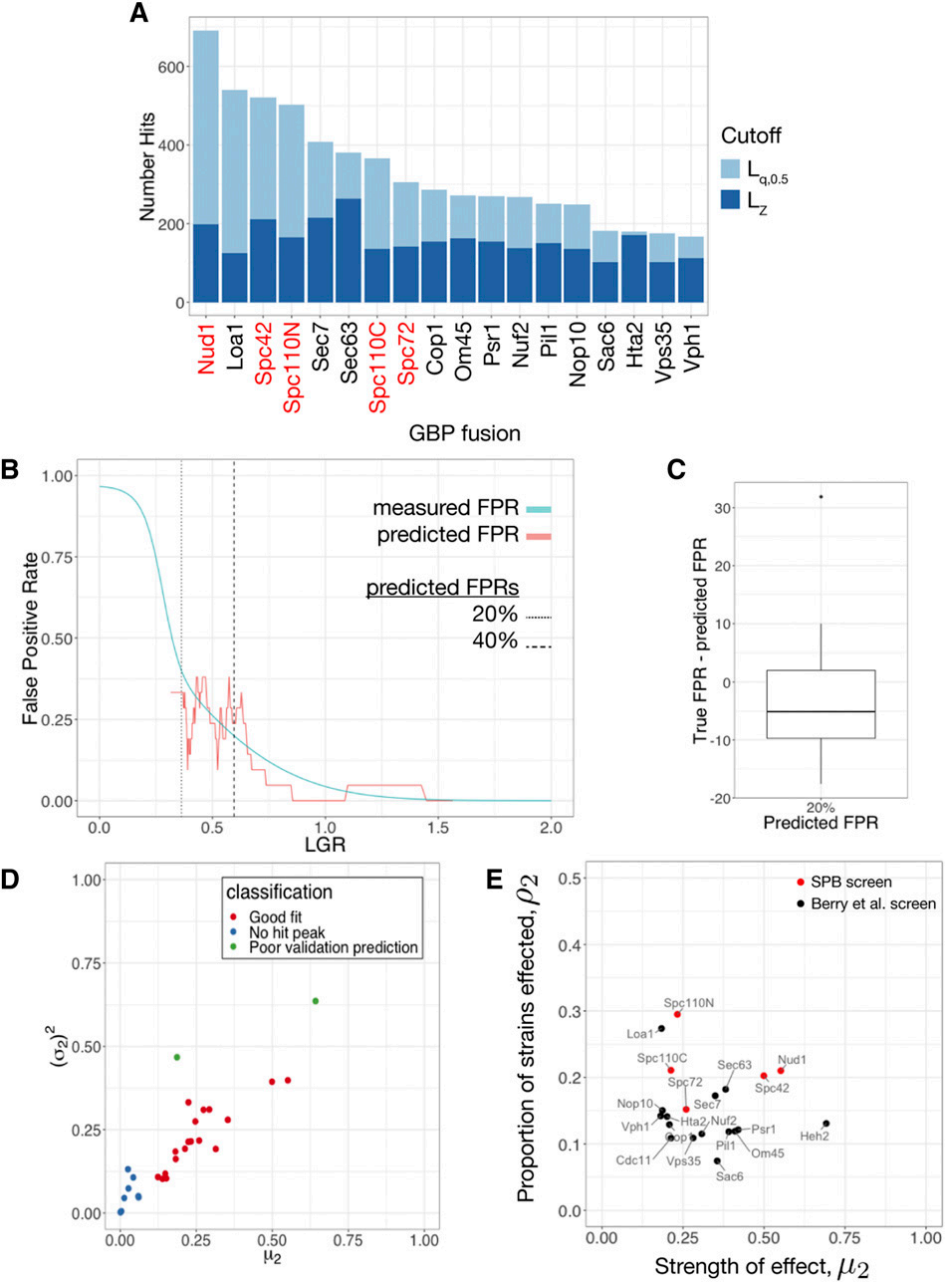
A: The number of hits by both Z-score ($L_{Z}$) and q(x) ($L_{q,0.5}$) cutoff for each of the screens where the mixture model was applicable. The q(x) cutoff has a higher dynamic range than the Z-score and is better able to distinguish screens with many hits. B: FPR prediction for the Spc72 screen. The FPR for the screen was predicted from the mixture model and this prediction is overlaid with estimates of the FPR using binned data from the validation screen. In this case, the predicted FPR was reasonably accurate, although the data are quite noisy. The points where the mixture model predicts 20% and 40% FPR are indicated with a dashed line. C: Box-and-whisker plot showing the difference between measured and predicted FPR at the point where the FPR is predicted to be 20% across the screens where the mixture model was applicable. This shows some bias, with the predicted FPR generally higher than the true FPR but generally achieving an accuracy around ±10%. D: Classification of mixture model fit for each of the 28 screens analyzed. The mean $\mu_{2}$ and variance ${\left(\sigma_{2}\right)}^{2}$ of component 2 are good indicators of the success of the model with very low means or high variances indicative of the lack of a hit peak or poor validation prediction respectively. E: Classification of screen based on fitted parameters calculated using the subset of GFP strains used in the SPB screen. Each of the screens for which the mixture model fit was appropriate are plotted according to the proportion of strains affected ($\rho_{2}$) and the average strength of these effects ($\mu_{2}$). The SPB screens Spc42 and Nud1 are positioned in the upper right portion of the graph, showing that a large proportion of proteins were sensitive to forced interaction with the SPB and these sensitivities caused significant growth defects.

Having determined that mixture models are a more appropriate statistical model than a normal distribution, we developed metrics to determine the significance of individual results and cutoffs to distinguish hits from non-hits. A typical approach in genome-wide screens is to calculate p-values based on a null model of the data. In the case of mixture models, identifying Component 1 as an empirical null model for the data allows for calculation of p-values, which may be adjusted for multiple hypothesis testing, for example by calculating FDR q-values (Benjamini and Hochberg 1995). However, in this context a more natural approach is to calculate the conditional probability of inclusion in Component 2. We define q(x) to be the probability of inclusion in Component 2 given a measured LGR of *x*. The point where q(x)=0.5 is the point where a strain with measured LGR *x* is equally likely to be in Component 1 or 2, and is therefore a logical point to place a cutoff. We define this point as $L_{q,0.5}$, while the point where a Z-transformation of the data has value 2 is $L_{Z}$. We found that $L_{q,0.5}$ always sat below the $L_{Z}$ but this effect was more pronounced in screens with more hits. Notably using Z-score as a cutoff limited the range of numbers of hits to 100-250. In contrast, using $L_{q,0.5}$ as a cutoff has a dynamic range of 100-700 hits (Figure 3A, Supplementary Table S1). This makes the mixture model approach a more effective tool than Z-score to distinguish between screens with many or few hits. We compiled a list of hits for each screen based either on $L_{Z}$ or $L_{q,0.5}$, depending on the success of the empirical Bayes approach (Supplementary File S4).

The top hits from genome-wide screens are commonly validated by repeating the screen either to verify key results or to establish metrics such as the False Positive Rate (FPR). Validation is undesirable as it requires further resources and, in some cases, may not be practical, so we developed a statistical method to predict the FPR. In the validation screens, 16 replicates were used as opposed to 4 in the original genome-wide screens. Furthermore, a hit was considered to be validated if the measured LGR was considered to be significant relative to GFP-free controls. Validation is considered to be a “gold-standard” for hit verification as it corresponds closely to other assays for growth defects such as spot tests. As the q(x) cutoffs were lower than the Z-score cutoffs we were concerned that they may not be reliable indicators of validation. Indeed, most of the q(x) cutoffs lay below the 40% FPR point at which Berry *et al.* (2016) stopped validating. It is worth noting that results that do not validate may still be reproducible and biologically interesting despite having relatively subtle effects on growth that are difficult to distinguish from the variability in wild type growth. Therefore, we developed a method to predict the likelihood of validation. Using the fitted mixture models, we developed a metric pV(x), representing the probability of validation for a strain with measured LGR *x*. pV(x) is generally successful at predicting the rate of validation in a screen (Figure 3B). For the 20 SPI screens which were fit well by the mixture models, the validation rate of 18 of these screens was predicted well by pV(x). The other two generally had very poor validation rates in general, making any kind of validation prediction unlikely to succeed. Plotting the variance and means of Component 2 for each of the SPI screens (Figure 3D) shows that both of these screens are outliers with very high variances. Therefore, we recommend that when using this approach, great care is taken when the variance of Component 2 is high. Comparison of specific points, for example 20% FPR, shows good predictive power (Figure 3C).

### The SPB is especially sensitive to forced relocalization

When we compared the SPI screens using SPB components with the previous screens using other structures throughout the cell, we noticed some key differences. Figure 3A shows that the SPB SPI screens are among the screens with the greatest number of hits, both by Z-score and q(x) cutoff. The fitted mixture models offer an additional way to understand this difference. Within a SPI screen, we may wish to distinguish between the case of a large proportion of strains being affected in a minor way and a smaller proportion of strains being very strongly affected. The fitted parameters $\rho_{2}$ and $\mu_{2}$ reflect the proportion of strains affected and the severity of these effects respectively. Plotting these two parameters together therefore provides a graphical way to compare screens. This is shown in Figure 3E (Supplementary Table S1), where we see that the SPI screens sit in the top-right region of the graph as they have high values of $\rho_{2}$ and $\mu_{2}$. In particular, Spc42 and Nud1 produce especially strong SPIs (high $\mu_{2}$), while the Spc110 screens produce weaker SPIs but with many different strains (high $\rho_{2}$). Spc72 is more midrange, possible reflecting the fact that in the S288C background, *SPC72* is a non-essential gene (Giaever *et al.* 2002). Notably, Loa1 has a high value of $\sigma_{2}$, Heh2 has a high value of $\mu_{2}$ and Sec7 and Sec63 sit near to Spc42 and Nud1. All four of these proteins localize to the ER, Golgi or nuclear membrane, suggesting that these regions specifically may be the most sensitive to forcible relocalization. It should be noted that the screens of Berry *et al.* used the full GFP library Huh *et al.* (2003), while the SPB SPI screens were performed with a smaller subset of 4,500 strains with confirmed GFP fluorescence Tkach *et al.* (2012). The parameters fitted to the screens of Berry *et al.* by the empirical Bayes method are not significantly affected by restricting the analysis to this subset of strains (Supplementary Table S2).

**Figure 4:**
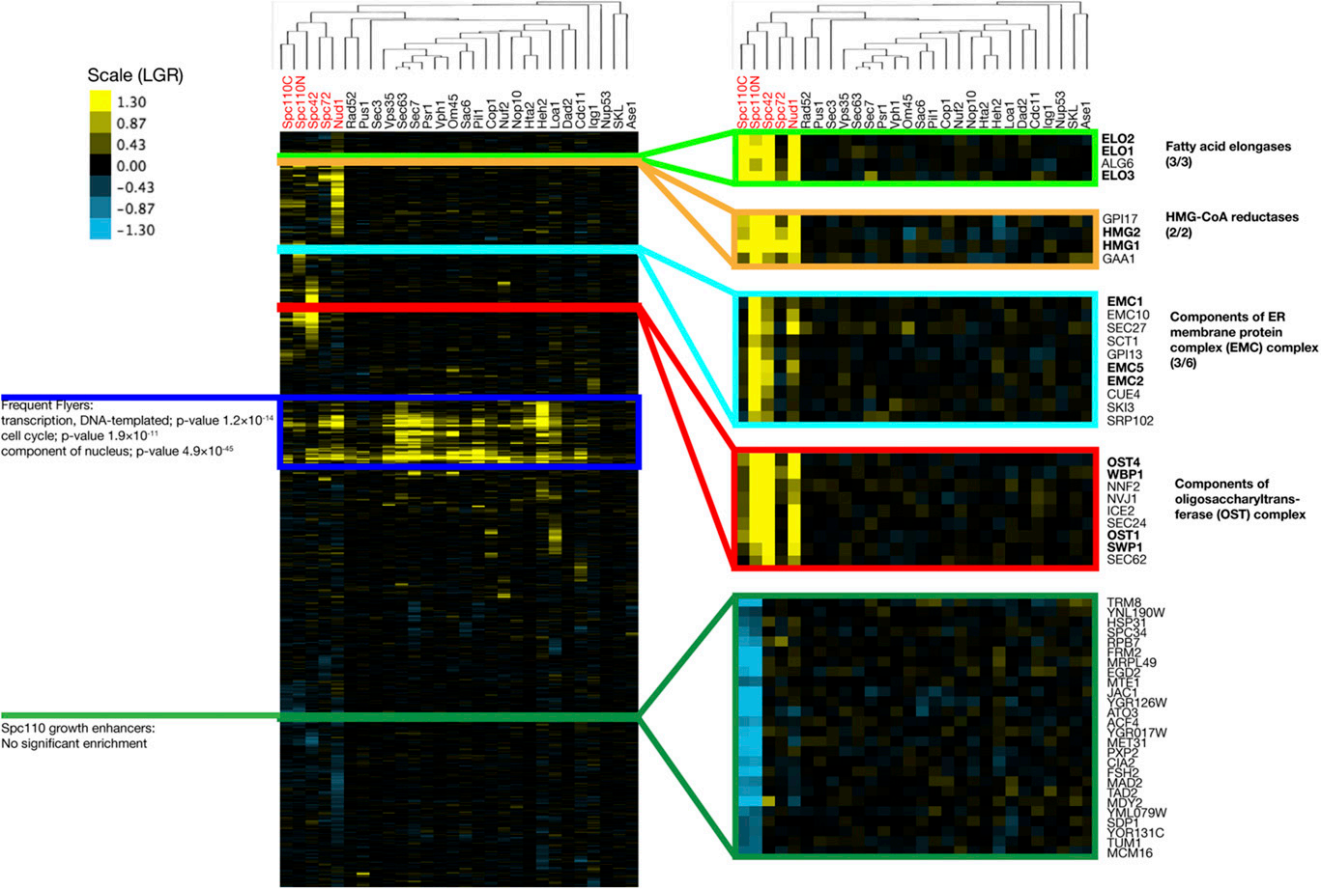
Cluster analysis of all 28 SPI screens used in this study. The data are clustered both vertically (by GFP strain) and horizontally (by screen, tree shown). The horizontal clustering tree shows that the SPB screen results are more similar to each other than the other screens. The vertical clustering identifies clusters of biologically related proteins with similar profiles of sensitivity to forcible relocalization.

We used hierarchical clustering to compare the SPB screens to the other SPI screens in the dataset (Figure 4). The data were clustered both vertically (by GFP strain) and horizontally (by screen). Clustering by screen shows the five SPB screens are more similar to each other than to other screens in the dataset, suggesting there is a characteristic set of proteins that are sensitive to forced localization to the SPB. A Venn diagram showing overlap between hits from the SPB SPI screens is shown in Supplementary Figure S1. Clustering the data by GFP strain identifies clusters of biologically related proteins with similar profiles of localization sensitivity, as previously reported (Berry *et al.* 2016). Clusters of proteins with SPIs with the SPB group together, for example, the fatty acid elongases Elo1, Elo2 and Elo3; and the two paralogs of HMG-CoA reductase Hmg1 and Hmg2. These clusters also link together members of protein complexes such as the ER membrane protein complex (EMC) and oligosaccharyltransferase complex (OST). The clustering identifies a group of proteins that appear to enhance growth when forced to interact with both termini of Spc110. This group is not significantly enriched for any GO terms, however, it does include Mad2, consistent with the idea that partial Mad2 perturbation may accelerate cell cycle progression (Barnhart *et al.* 2011). However, we found that growth enhancers were unlikely to reproduce their behavior in validation screens. The vertical clustering also identifies a collection of proteins that are sensitive to forcible relocalization to all or most parts of the cell. This group of proteins, known as ”frequent flyers”, are enriched for transcription factors and nuclear proteins.

**Figure 5:**
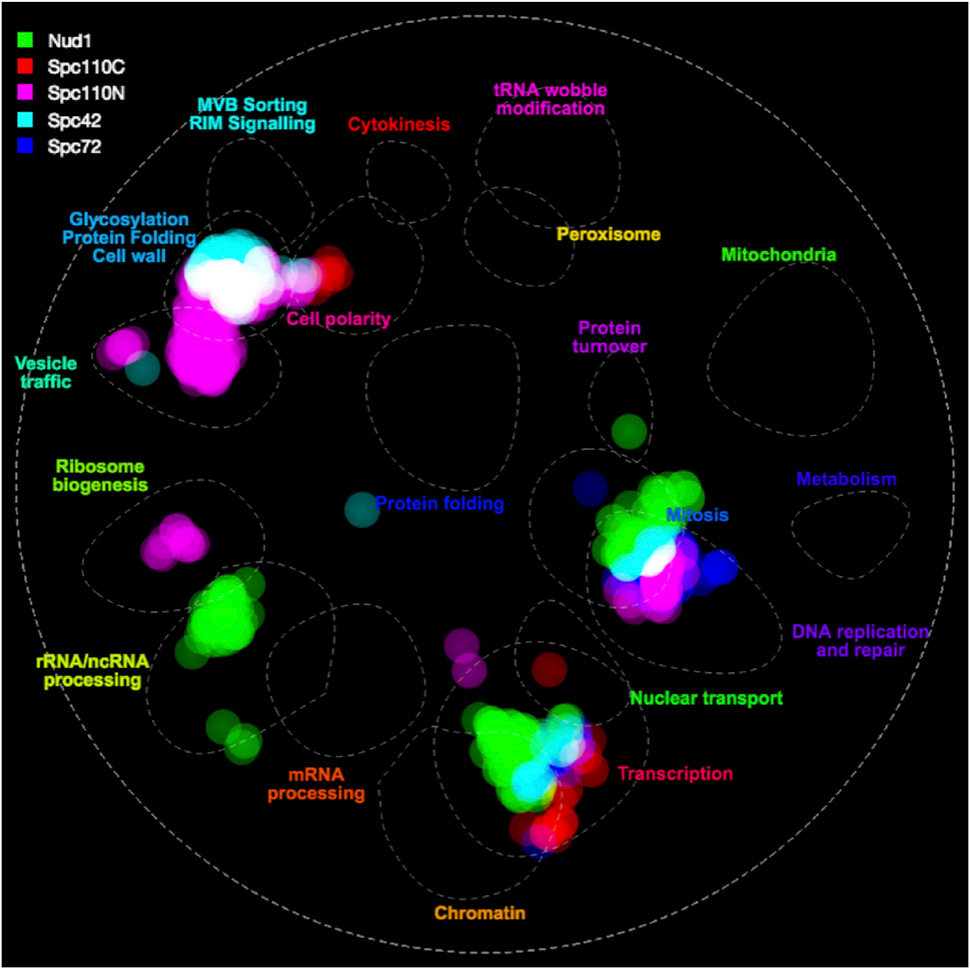
SAFE enrichment of hits from SPB SPI screen, visualized using TheCellMap.org. The *S. cerevisiae* genetic interaction network was clustered by density, identifying highly dense regions of space corresponding to shared function. Regions of space containing high densities of hits from each screen are highlighted accordingly showing visually which processes the hits from the screens are related to.

In order to understand which kinds of proteins are sensitive to forced relocalization to each part of the SPB, we used the online tool thecellmap.org (Usaj *et al.* 2017) to perform spatial analysis of functional enrichment and visualize the results (Figure 5). SPB SPIs cluster together in the region of the network containing mitotic regulators and transcription factors. Additionally, there is a cluster of hits from the Spc110 and Spc42 screens in the region containing proteins involved in cell wall, glycosylation and protein folding. There are further clusters for individual screens including Spc110 N-terminus clusters in ribosome biogenesis and vesicle traffic regions and a cluster of Nud1 hits in the rRNA/ncRNA processing region.

**Figure 6:**
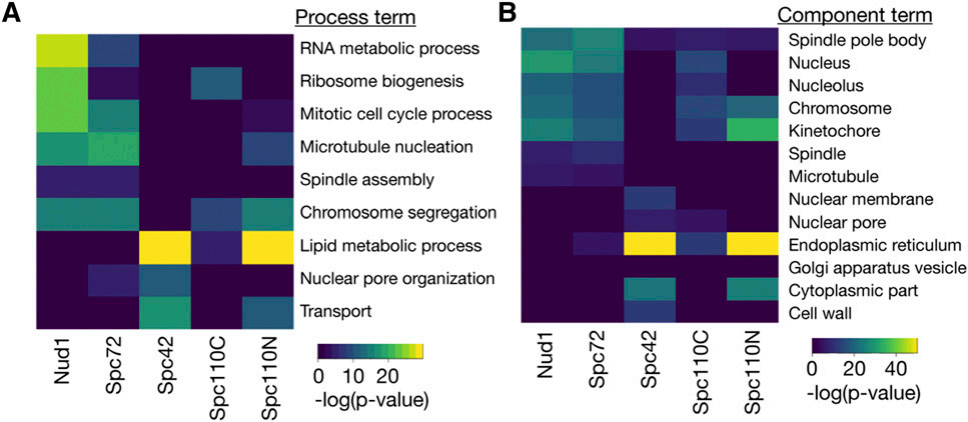
GO analysis of SPB SPI screens, performed using the entire, ranked dataset. A: Heatmap of process GO analysis, dark blue tiles represent no significant enrichment while the lighter colors represent significant enrichment, with warmer tones representing higher p-values. B: Heatmap of component GO analysis.

We performed Gene Ontology (GO) analysis of the ranked LGRs for each of the screens using the GOrilla tool (Eden *et al.* 2009). Rather than using a list of hits from the SPI screen, we used the entire ranked list for each dataset, negating the need for a cutoff LGR. Heatmaps of significant enrichments are shown in Figure 6 (Supplementary File S5). The screens with Spc42, Spc110C and Spc110N fusions were all significantly enriched for proteins involved in lipid metabolic process and proteins from the ER. In particular, there was significant enrichment for proteins involved in biosynthesis of sterols, sphingolipids and those involved in fatty acid elongation (Supplementary Figure S2). The position of the SPB, embedded within the nuclear membrane (Jaspersen and Winey 2004), suggests that these growth defects may result from disregulation of nuclear membrane composition. Furthermore Witkin *et al.* (2010) found that deletion of *SPO7*, a regulator of phospholipid biosynthesis, could partially suppress the monopolar phenotype of mutations in *MPS3*, suggesting that the membrane environment can impact on SPB duplication. We also found that the screens with Nud1, Spc72 and Spc110N, the proteins located closest to the sites of microtubule nucleation, were enriched for proteins involved in the process of microtubule nucleation. These findings suggest that targeting these proteins artificially to the SPB can induce growth defects, possibly due to problems with spindle formation or nuclear positioning. An intriguing result is the finding that all screens, except Spc42, were enriched for proteins involved in chromosome segregation and components of the chromosome and kinetochore; there is evidence for example from yeast-two-hybrid screens that kinetochore proteins physically interact with SPB components (Wong *et al.* 2007). It is worth noting that these phenotypes may simply represent disruption of these structures by removal of the protein, although these proteins were not frequent flyers. Nud1 and Spc72 are thought to act as a signaling scaffold for proteins in the Mitotic Exit Network pathway (Scarfone and Piatti 2015) and screens with these proteins were enriched for mitotic cell cycle proteins. Finally, we found that the Spc42 screen was enriched for proteins involved in nuclear pore organization as well as subunits of the nuclear pore. Intriguingly, some of these findings overlap with known genetic interactions, for example deletion of *NUP157* suppresses the *spc42-11* mutation (Witkin *et al.* 2010), while we found that tethering Nup157 to Spc42 lead to a growth defect. Due to the proposed link between SPB duplication and insertion and the nuclear pore (Rüthnick and Schiebel 2018) we investigated these results further.

### SPIs with the SPB lead to SPB overduplication

We investigated whether we could detect any SPB duplication phenotype caused by forcible localization of proteins to the SPB. We screened 80 query proteins that we suspected would cause defects in SPB duplication against the Spc42-GBP-RFP fusion. These proteins included hits from our screen and other proteins known to function in the same pathways or complexes. For example, we identified Nsp1, a nucleoporin, in our screen and included other nuclear pore components such as Nup170, which was not a SPI in our original screen. Cells were imaged using fluorescence microscopy and the number of Spc42-GBP-RFP foci were counted. In strains expressing membrane or pore proteins tagged with GFP we observed recruitment of Spc42-GBP-RFP to these regions, however small regions with relatively high RFP signal were observed and these were counted as foci. We observed cells with 3 or more RFP foci in eleven different strains (Table 2, Supplementary Table S3). In some cases, a single red focus was observed in large budded cells, however the slow-folding nature of RFP prevented us from ruling out the possibility of further SPBs that are unmarked by mature RFP.

**Table 2:** Proteins identified in the microscopy screen for proteins that induce extra SPBs when forcibly relocalized to the SPB. ${}^{\text{*}}$ YJL021C overlaps the originally identified YJL020C ORF and so has been merged into YJL020C (Brachat *et al.* 2003), however the GFP strain shows a punctate fluorescent signal. Database locations were accessed from yeastgenome.org/

Protein	Database Location	Screen LGR	Retest LGR
Apq12	ER	1.58	1.46
Crm1	Nucleus	1.23	0.54
Nic96	Nuclear Periphery	1.06	1.20
Nsp1	Nuclear Periphery	0.91	0.36
Nup133	Nuclear Periphery	0.23	0.27
Nup170	Nuclear Periphery	0.19	0.33
Pom34	Nuclear Periphery	0.46	0.77
YDL121C	ER	2.40	1.83
YJL021C${}^{\text{*}}$	Punctate	0.74	0.95
YPR071W	ER	0.42	0.82
YPR114W	ER	2.67	2.01

**Figure 7:**
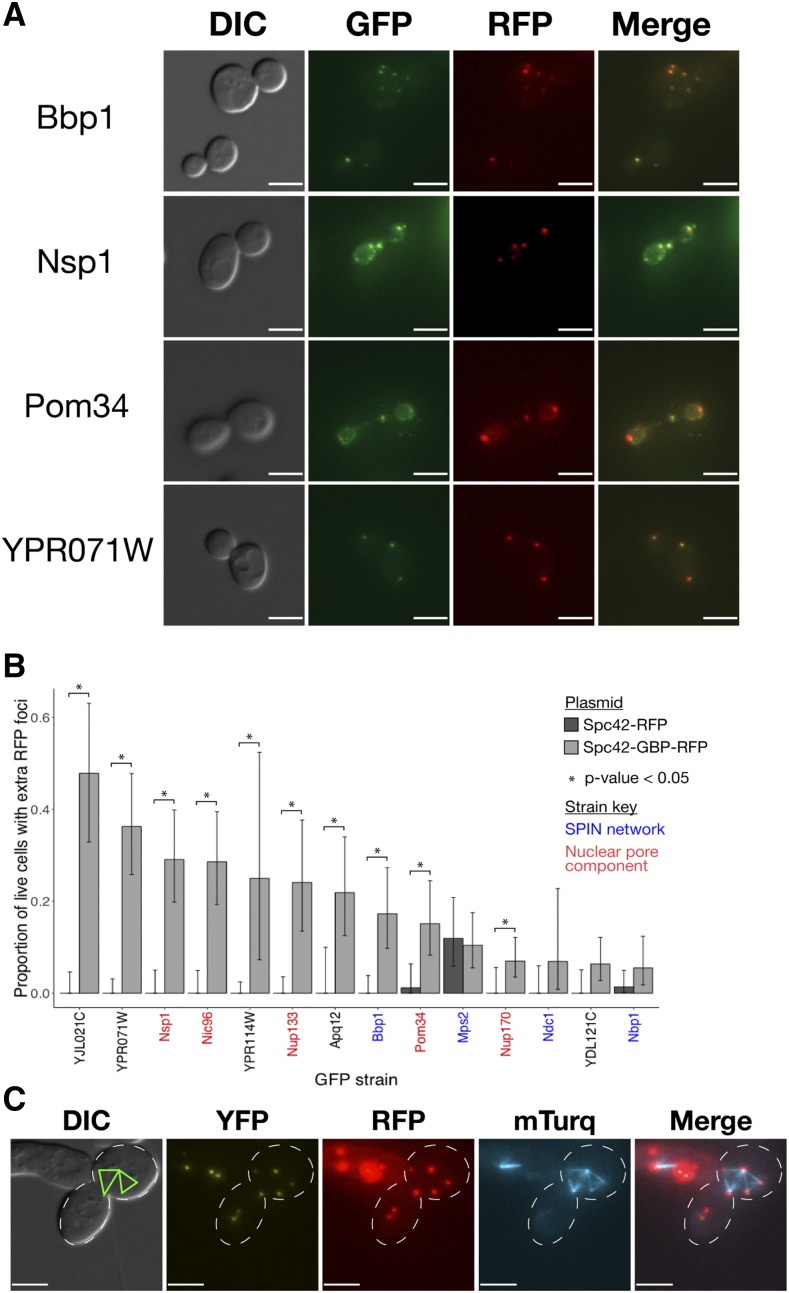
Extra RFP foci phenotype. Plasmids encoding either Spc42-GBP-RFP or Spc42-RFP were directly transformed into the GFP strains identified in the screen for SPB number aberrations and imaged. A: Representative images of Bbp1, Nsp1, Pom34 and YPR071W -GFP strains expressing Spc42-GBP-RFP from a plasmid, each showing more than two RFP foci, interpreted as indicative of overduplication of SPBs. All scale bars are 5μm. B: Quantification of the microscopy analysis, with key proteins highlighted. Three images were captured for each strain and the percentage of living cells showing more than two RFP foci was calculated. Error bars show 95% confidence intervals calculated with the Clopper-Pearson exact method; p-values were calculated using Fisher’s exact test. C: Multipolar spindle morphology in the Spc42-GBP-RFP Bbp1-YFP strain, the spindle is labeled by mTurq-Tub1. Green lines indicate the observed spindle morphology, from the mTurq-Tub1 signal, relative to the cell morphology observed in DIC. Scale bars are 5μm.

Screening cells directly from the SPI screen meant that limited number of cells were available to image in slow-growing SPI strains. Therefore, we directly transformed these strains, alongside the four members of the SPIN network, with the Spc42-GBP-RFP plasmid as well as a control plasmid expressing Spc42-RFP. We were able to establish colonies of all strains except Crm1-GFP. Using these strains, we imaged larger quantities of these cells (Figure 7A, Supplementary Figure S3). We detected extra red foci in each of the strains expressing Spc42-GBP-RFP and quantified the proportion of cells expressing this phenotype (Figure 7B, Supplementary Table S4). In most of the GFP strains transformed with the control plasmid we did not detect additional RFP foci, however to our surprise we did observe additional foci in the Mps2-GFP strain and, to a much lesser extent, the Nbp1-GFP and Pom34-GFP strains. Forced recruitment to the SPB of all proteins gave rise to statistically significant proportions of cells containing additional RFP foci, with the exception of Mps2, Ndc1, YDL121C and Nbp1. Notably, we found that the strength of growth defect as measured by the LGR was not a strong indicator of the frequency of extra red foci, suggesting the growth defect does not arise entirely from this phenotype. Note that the protein denoted by its ORF, YJL021C, is included in these results however this ORF was determined to overlap *YJL020C* Brachat *et al.* (2003) meaning the GFP product in this strain is likely not a simple N-terminal fusion. Furthermore, the GFP strain shows a punctate fluorescent signal, meaning the extra red foci in these cells may represent relocalization of Spc42-GBP-RFP to YJL021C-GFP foci. These results suggest that the forced interaction of these proteins with the SPB results in aggregates of the Spc42 protein that may indicate extra SPBs. In order to gain a further insight into this phenotype, we constructed strains expressing mTurq-Tub1 and Bbp1-, Nup133-, Nup170- and YJL021C-YFP respectively, and transformed them with the Spc42-GBP-RFP plasmid. YFP has enough homology to GFP that it interacts strongly with GBP but the fluorescent signal is distinguishable from mTurq, allowing for identification of protein localization in three colors. Analysis of spindle morphology in the Bbp1-YFP strain revealed mTurq-Tub1 signal between multiple red foci, and in some cases showed a multi-polar spindle phenotype (Figure 7C and Supplementary Figure S4), suggesting the additional red foci observed in this strain represent SPBs and not simply aggregates of Spc42-GBP-RFP. Data from the Nup133- and YJL021C-YFP strains also indicate that the red foci represent SPB-like structures that are capable of nucleating microtubules (Figure S5). In Nup170-YFP cells with multiple red foci, we observed only two foci associated with mTurq-Tub1 signal, suggesting the red foci in this strain may not represent functional SPBs.

## Discussion

### Analysis of SPI screens

Z-transformations are a common tool to analyze genome-wide screens, however their underlying assumption of normally distributed data means they can produce unreliable results, especially in screens identifying many hits. We developed an empirical Bayes approach to address the shortcomings of Z-transformations. Rather than use the Z-score to test significance we introduced a cutoff based on the probability of inclusion, which is more effective in discriminating between screens with many hits. Notably, some hits in the Spc42 screen, such as Nup133, had LGRs which would have been considered insignificant according to the Z-score cutoff, but showed a distinct phenotype in further assays. This shows that the lower cutoffs we propose can still be biologically relevant. Additionally, we developed a method to predict the validation rate of the screen with reasonable accuracy. These two metrics provide alternative viewpoints on the significance and strength of a given result within a screen. Furthermore, bimodal normal mixture models have 5 independent parameters, allowing for more effective parameterisation of the distribution than the 2 parameters of a single normal distribution. These parameters can be used to compare the distribution of results from different screens, providing a way to understand the differences between SPI screens. Previous analysis of cell-wide SPI screens concluded that only a small proportion of proteins are sensitive to forced relocalization however our re-analysis of this data and the inclusion of the SPB in this dataset suggests that some regions of the cell are far more sensitive to forced relocalization of proteins across the proteome. The empirical Bayes approach offers the most significant benefits in the cases where Z-transformations are least appropriate: when analyzing screens with large numbers of hits. However, we did find that when the fitted standard deviation of Component 2 was too large, the screens validated poorly and the mixture model was inaccurate.

Mixture models allow for a quick and easy way to effectively parameterise screening distribution data, however they cannot provide perfect prediction with imperfect data. If greater levels of precision or reproducibility are necessary, further modifications to the experimental procedures would be required. Zackrisson and colleagues found significant variation in growth rates of colonies across a single plate and recommend local normalization of colony size to account for these effects to improve reproducibility (Zackrisson *et al.* 2016). Baryshnikova and colleagues found that “batch” effects, caused by subtle differences in, for example, media composition or incubator temperature, between plates grown at different times, caused significant variation in colony sizes (Baryshnikova *et al.* 2010). While all plates in a SPI screen are generally grown concurrently, the validation screens were performed afterward, once analysis of the screen has been performed. This may explain the high FPRs in validation of some screens. Baryshnikova and colleagues propose using linear discriminant analysis to compute “batch signatures” which could be used to limit batch effects. Finally, the precision of measurements could be improved by a methodology that directly correlates growth measurement to rate, for example by calculating a growth curve using automated scanning of plates at regular intervals (Zackrisson *et al.* 2016).

### SPB overduplication

Our current understanding of the SPB duplication cycle of *S. cerevisiae* is that alternating activities of the CDK-cyclin complex and Cdc14 phosphatase are responsible for once-per-cycle duplication of the SPB (Rüthnick and Schiebel 2018). This model suggests that SPB duplication is initiated while Cdc14 activity is at its peak but may not be completed until CDK activity increases and Cdc14 activity decreases later in the cell cycle. A reasonable prediction would be that forced recruitment of Cdc14 to the SPB would induce overduplication of SPBs, however we found that forced recruitment of Cdc14 only produced a growth defect with the outer plaque component Nud1 and we did not observe evidence of SPB overduplication when Cdc14 was recruited to any part of the SPB (unpublished observations). It is worth noting that a previous SPI screen found that the combination of Cdc14-GBP and Spc42-GFP induced a growth defect that depended on the phosphatase activity of Cdc14 Ólafsson and Thorpe (2015), however when the tags were switched in the screen reported here, we found no growth defect.

It has been proposed that the SPB satellite is inserted into the nuclear membrane using molecular machinery that is responsible for nuclear pore complex (NPC) insertion (Rüthnick and Schiebel 2018). We screened NPC and SPIN proteins for SPIs with Spc42 and used fluorescence microscopy to count Spc42 foci in these strains. We found evidence that forcible recruitment of the SPIN component Bbp1 to the SPB induced formation of additional Spc42-GBP-RFP foci, which were capable of nucleating microtubules. We note that the ability of the extra SPB foci to nucleate microtubules does not necessarily mean that these foci represent canonical SPBs, a possibility which could be explored with further fluorescent SPB markers. We also found that the NPC components Nsp1, Nic96, Nup133 and Pom34 produced extra foci and that in the case of Nup133, these foci colocalized with tubulin. It is interesting that the deletion of either of two of the genes coding for these proteins, *NIC96* and *POM34*, were identified as suppressors of SPB duplication defects caused by *mps3-1 spo7*Δ mutation (Witkin *et al.* 2010). This finding suggests that in their wild type localization, these proteins inhibited SPB duplication, possibly by competing for binding partners, whereas our data suggests that when forced to the SPB, these proteins can induce the overduplication of SPBs. Additionally, we found evidence that the, as yet, unclassified proteins encoded by YJL021C, YPR071W, YPR114W and YDL121C as well as Apq12 similarly induce extra Spc42 foci indicative of SPB overduplication. There are several interpretations of these findings, which may apply to some or all of the phenotypes observed. First, it is possible that the RFP foci observed may represent aggregates of Spc42-GBP-RFP that do not contain other SPB proteins or function as MTOCs. It is worth remarking that in a systematic study of localization of target and query proteins, a small proportion were found to localize to a region of the cell where neither would localize in wild type cells (Berry *et al.* 2016). Second, it may be that forced recruitment of these proteins induce SPB overduplication through the documented SPB duplication pathway. This would require detachment of this process from the once-per-cycle regulation via CDK-cyclin and Cdc14. This could be explained if some aspects of this process were initiated by the presence of these proteins at the SPB, which in wild-type cells was induced by CDK or Cdc14 activity. Finally, it may be the case that targeting Spc42 to other structures in the cell, especially the NPC, can lead to the creation of *de novo* SPBs. The current model of SPB duplication suggests that SPBs assemble from a satellite formed of Spc42, Nud1, Cnm67 and Spc29 (Fu *et al.* 2015). It may be that a small amount of Spc42 is recruited to the NPC in these strains, seeding new SPBs in a manner completely distinct from regular SPB duplication. Witkin *et al.* (2010) proposed an *MPS3* independent SPB duplication pathway and it may be this or some other pathway that is responsible for this phenotype.

Further work is required to distinguish these models, in particular, assessment of the foci for presence of other SPB proteins and functionality of the foci as MTOCs is required to confirm them as real SPBs. If SPBs are created *de novo* we would expect that these strains would lose the requirement for proteins with an essential role in SPB duplication, such as Cdc31 (Rüthnick and Schiebel 2016). Many mutants have been identified that fail to duplicate their SPBs, for example the original MPS (Mono-Polar Spindle) genes (Winey *et al.* 1991) however there are fewer cases of genetic perturbations that lead to SPB overduplication. Overexpression of Cdc5 or mutations to Aurora kinase, Ipl1, have been shown to cause overduplication of SPBs in meiosis (Shirk *et al.* 2011). In mitosis, deletion of all *CLB* genes (Haase *et al.* 2001) and overexpression of Cdc5 (Song *et al.* 2000) have been demonstrated to induce SPB overduplication however the pleiotropic effects of these mutations may limit their use as model systems to study multi-polar spindles. The *sfi1-C4A* mutation (Avena *et al.* 2014), causes SPB overduplication without perturbing key cell cycle control mechanisms however this strain also exhibits an SPB seperation defect meaning additional SPBs are kept tethered to the original SPB, preventing formation of multi-polar spindles. Therefore, the multi-polar spindle phenotype caused by forced association of Bbp1 and Spc42 may offer a useful system to investigate physiological responses to multi-polar spindles.
